# Exploring the Nutritional and Anti‐Nutritional Composition of Traditionally Fermented *Qocho* From Widely Cultivated Enset (*Ensete ventricosum*) Landraces in Central Ethiopia

**DOI:** 10.1002/fsn3.70216

**Published:** 2025-04-30

**Authors:** Tesfaye Dilebo, Ashagire Zewdu

**Affiliations:** ^1^ Department of Natural Science Hossana College of Education Hossana Ethiopia; ^2^ Centre for Food Science and Nutrition Addis Ababa University Addis Ababa Ethiopia

**Keywords:** anti‐nutrients, bioavailability, Enset, minerals, physiochemical, proximate

## Abstract

Enset is a multipurpose perennial root crop primarily used as a staple food for over 25 million people in Ethiopia. Despite the existence of various landraces, little information is known about their nutritional profile. Thus, the aim of this study was to determine the nutritional and anti‐nutritional contents of *qocho* from the eight widely cultivated landraces. Traditionally fermented *qocho* samples were milled individually into a fine powder and analyzed by applying standard food procedures. The mean proximate component (%) varied in moisture value from 59.0–66.5, crude protein (1.75–3.15), crude fat (0.14–0.73), crude fiber (2.25–5.39), and total ash (1.2–2.4), whereas the total carbohydrates came to 89.74–94.64, and gross energy was 370.69–387.97 kcal/100 g. The mean contents of minerals (mg/100 g) ranged: calcium (80.17–110.60), potassium (90.35–157.14), magnesium (14.37–16.35), phosphorus (10.84–40.19), sodium (7.41–8.35), iron (4.08–6.71), and zinc (0.39–0.73) on a dry weight basis. The mean anti‐nutritional values (mg/100 g) for oxalate, tannin, and phytate ranged from 6.26–9.39, 5.04–32.05, and 74.28–141.19, respectively. This showed that the *qocho* samples contain low contents of anti‐nutritional factors. Regarding molar ratios, phytate to calcium, phytate × calcium to zinc, and oxalate to calcium were shown below the critical values. Overall, the *qocho* of the analyzed enset landraces had a considerable variation in nutritional profile. Therefore, continued identification is crucial to distinguish the enset landraces with more nutritious *qocho* for local and regional consumption as well as to sustain enset cultivation for food security.

## Graphical abstract

The *qocho* samples of each landrace contained various amounts of proximate, minerals, anti‐nutritional, and molar ratios. All the analyzed samples showed a low level of anti‐nutritional factors. *Qocho* has a promising future in terms of food security and nutrition.

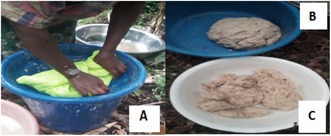

## 1 Introduction

Food and nutrition insecurity has been a serious problem in sub‐Saharan Africa due to climatic extremes and other factors (Yimer et al. 2023), and periodic droughts are not a recent occurrence in the Horn of Africa (Kassaye et al. 2021). Therefore, it has been suggested that in order to combat food insecurity and malnutrition, diet diversification and the search for alternate food sources be undertaken (Yimer et al. 2023). In many regions of the world, especially in developing nations, plant‐based products serve as the primary source of food for humans (Castro‐Alba et al. 2019). In these nations, roots and tubers crops are important to agriculture and contribute to food security (Neela and Fanta 2019). Enset is a multipurpose root crop mainly used for human and animal food, medicinal uses, fiber production, and fuel (Brandt et al. 1997; Tsegaye and Struik 2002; Dilebo, Feyissa, Asfaw, and Zewdu 2023a). Enset ( *Ensete ventricosum* (Welw.) Cheesman) is one of the seven species of the genus Ensete belonging to the Musaceae family within the order Zingiberales (Borrell et al. 2019). In the central, south, and southwest parts of Ethiopia, it is an indigenous agricultural system that may have developed characteristics that offer resilience approaches to handling environmental change and mitigating food insecurity (Matewos 2019; Chase et al. 2022). Hence, enset‐based agriculture is a good illustration of a local agricultural system that has reportedly adapted to withstand climate change (Pijls et al. 1995; Brandt et al. 1997; Satori et al. 2022).

Enset farming is a direct strategy for assisting individuals in achieving independent livelihood security (Senbeta et al. 2022), and for the more than 25 million residents in different administrative regions of Ethiopia, it is a major staple crop (Yemataw et al. 2016; Borrell et al. 2020). Due to its role in food security, enset is known as “the tree against hunger” by its growers and consumers (Brandt et al. 1997). Enset may be harvested all year round, thus ensuring a constant supply of nourishment. The major food types from enset are *qocho* (prepared through fermentation), *bulla* (produced through extraction), and *amicho* (boiled corm) (Tsegaye and Struik 2002).

The majority of enset landraces are grown primarily for their *qocho*, or *bulla*, which can be processed through fermentation (Negash 2001; Tsegaye and Struik 2002; Daba and Shigeta 2016; Yemataw et al. 2016; Borrell et al. 2020; Dilebo, Feyissa, and Asfaw 2023b). According to the reports of Daba and Shigeta (2016), enset landraces are classified into two main groups, namely, the *qocho* types and the corm types. However, *qocho* is the most commonly consumed and largely produced enset fermented product (Maryo et al. 2014; Olango et al. 2014; Dilebo, Feyissa, and Asfaw 2023b). Moreover, its abundance, relative cheapness, and long‐term storable carbohydrates are important for Ethiopia, where a considerable number of the population is still undernourished (Bosha et al. 2016; Borrell et al. 2020).

Prior studies have suggested that enset products are a good source of minerals and carbohydrates. Similarly, Atlabachew and Chandravanshi (2008) and Debebe et al. (2012) reported that *qocho* contains a sufficient amount of different minerals for human requirements compared with cereal flours. However, the production and nutrient content of enset vary according to the variety of landraces, age of the plants, management system, and environmental conditions (Bosha et al. 2016; Daba and Shigeta 2016) and the types of enset products used (Dilebo, Feyissa, Asfaw, and Zewdu 2023c).

Considering the fact that enset plants are more varied in Ethiopia in terms of cultivator's choice criteria, farming pattern, and consumption level, little scientific research has been conducted on the nutritional and anti‐nutritional components of widely farmed and often used enset landraces. The scope of information available regarding the *qocho* of enset is more limited to an overall enset crop or an unmentioned small figure of landraces with limited parameters. Consequently, it is imperative to comprehend the nutritional profile of these multipurpose and diverse crops for sustainable consumption. Furthermore, this will serve to enhance food safety and the long‐term utilization of the enset crop. In view of these circumstances, this study was initiated to ascertain the nutritional and anti‐nutritional constituents of the *qocho*, which was processed from eight distinct enset landraces that are widely consumed and locally favored.

## 2 Materials and Methods

### 2.1 Sample Collection

*Eight healthy enset landraces*: *Agade*, *Anchire*, *Gimbo*, *Hayiwona*, *Hiniba, Qiniwara*, *Separa*, and *Sisqella* were designated according to their appropriateness for *qocho* preparation and consumption and other uses recognized by local knowledgeable senior female farmers. Moreover, the chosen landraces were popular, frequently consumed, widely distributed, and accustomed to having constant local names throughout the Hadiya and other neighboring zones in central Ethiopia (Yemataw et al. 2016; Dilebo, Feyissa, Asfaw, and Zewdu 2023a). In enset‐cultivating regions of Ethiopia, female farmers are entirely responsible for activities, such as harvesting, processing, storing, preparing, and marketing the enset products (Brandt et al. 1997; Tsegaye 2002; Dilebo, Feyissa, Asfaw, and Zewdu 2023a; Dilebo, Feyissa, and Asfaw 2023b). Traditionally, the landraces were propagated vegetatively for several generations and then transferred three times prior to being permanently cultivated in fields. Furthermore, no chemical fertilizers were used to nourish the landraces. To reduce any variability that could arise from soil fertility, management practices, and other environmental factors, all of the chosen landraces were gathered from a single farmer's homegarden at the age of 7 in the Lemmo district of the Hadiya Zone, Central Ethiopia region. The district, situated at 2105–2510 m.a.s.l., experiences typical mean temperatures between 10.38°C and 22.57°C as well as 950–1540 mm of rainfall, exhibiting a bimodal distribution. Most soil types in the district are vertisols, with pH values between 5 and 6, according to reports from the Agricultural Office.

### 2.2 Sample Preparation

**FIGURE 1:**
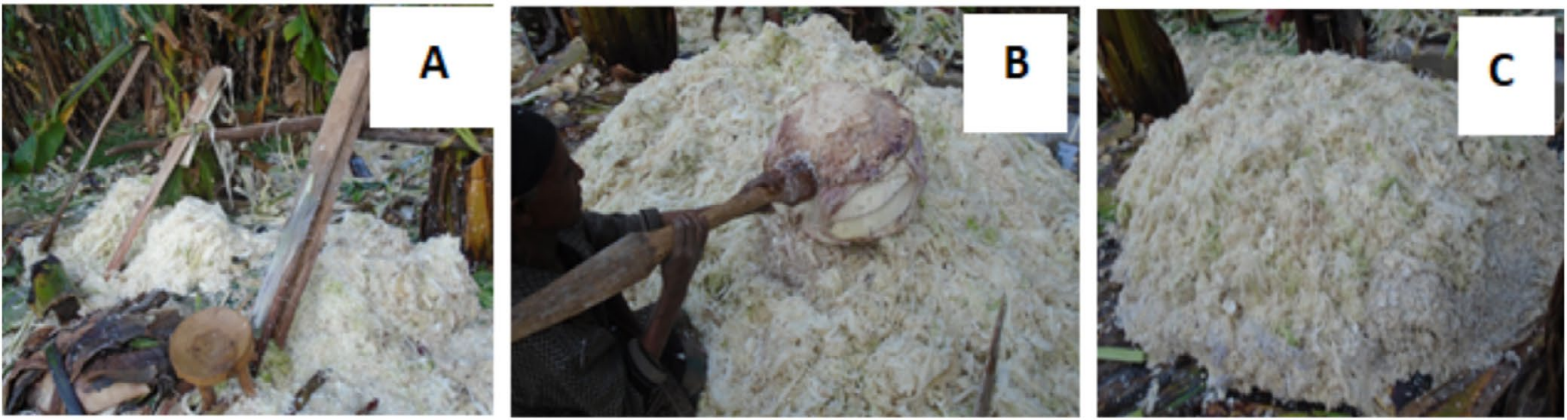
Different harvesting steps for *qocho* production: (A) Scraping of pseudostem (B) Pulverizing of corms (C) mixed mass of unfermented *qocho*.

The landraces were harvested and prepared individually following the traditional procedures of *qocho* preparation by knowledgeable senior female farmers during the semi‐dry season in late December 2020 (Figure 1).

**FIGURE 2:**
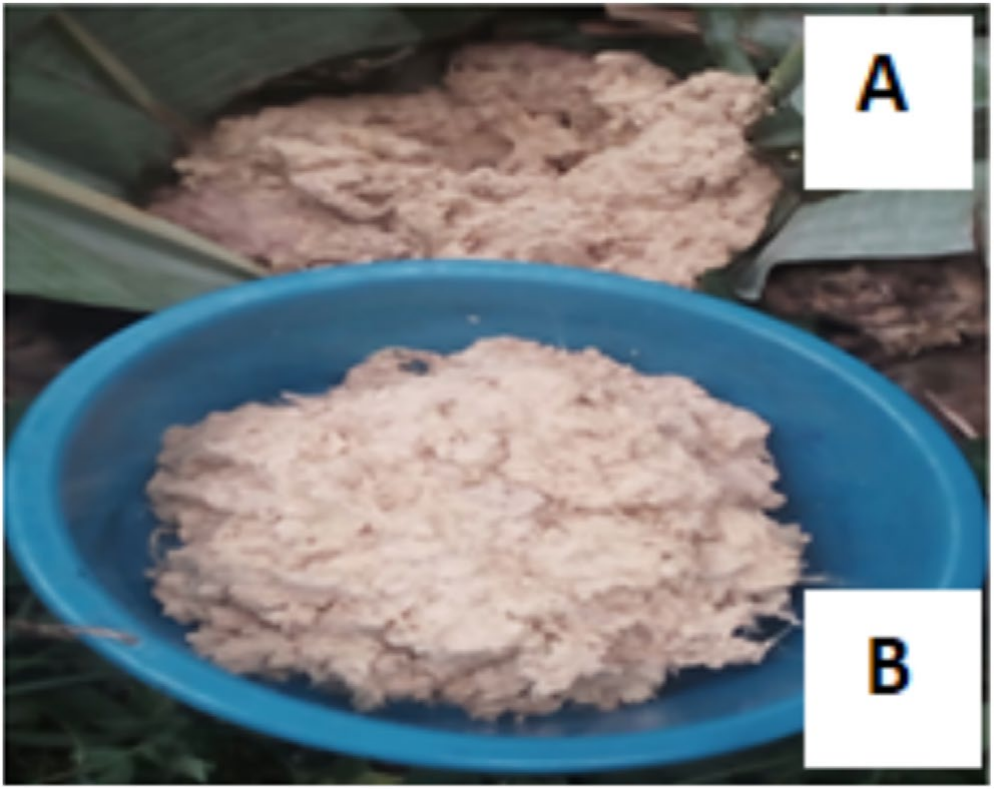
Traditionally fermented *qocho*: (A) fermented *qocho* in pit, (B) *qocho* ready for squeezing.

All the fermentation processes were carried out on the enset farm in the homegarden of the farmer for 3 months using the traditional pits (Figure 2).

**FIGURE 3:**
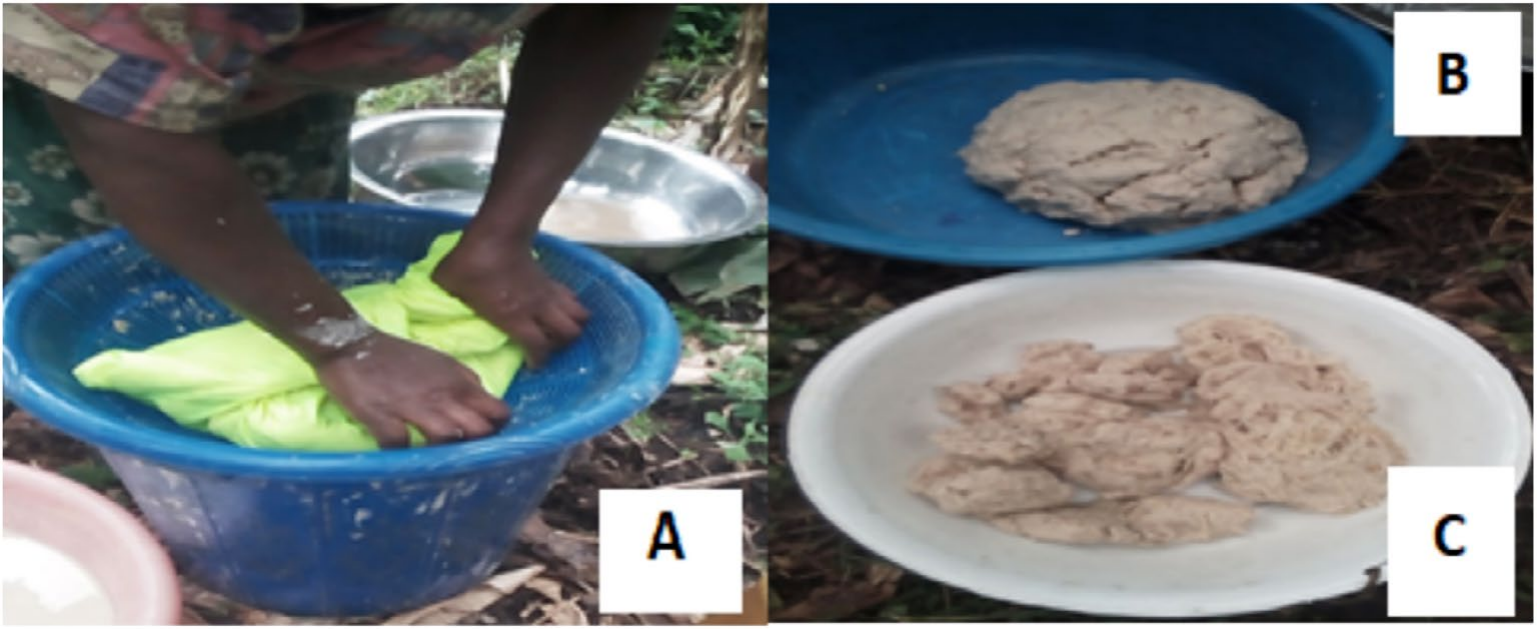
Different processing steps: (A) Squeezing for reducing excess water content, (B) *qocho* ready for different utilization, (C) unfermented wastes.

For the laboratory analysis, a small portion of the fermented *qocho* was taken out of the pit and independently dissolved in clean water, followed by the sieving out of the unfermented parts, then placed in the clean porous nylon sacks to remove excess moisture by squeezing out the liquid from them (Figure 3).

About 2 kg of each squeezed sample of *qocho* were packed in zipped polyethylene bags, preserved in an ice box to avoid moisture loss, and transported to the Center for Food Science and Nutrition Laboratory of Addis Ababa University. Each of the landrace *qocho* samples was measured for moisture content prior to drying. Each sample of *qocho* was placed on an aluminum foil tray and desiccated in an oven (DHG 9055A) at 50°C overnight before analysis to undergo further laboratory testing with minor modifications (Tuffa 2019). Each dried sample of *qocho* was finely ground in an electric grinder and then filtered through a 0.425 mm sieve. Subsequently, the milled *qocho* samples were packed in zip‐lock polyethylene bags and stored at 4°C to reduce heat accumulation until needed for proximate, mineral, and anti‐nutritional analyses.

### 2.3 Analysis of Proximate Composition

Each analysis, with the exception of the moisture, was done using a dry weight basis. Therefore, once the samples were dried and ground into flour, their dry matter contents were ascertained in triplicate.

The proximate constituent of the *qocho* samples was conducted following the Association of Official Analytical Chemists (AOAC 2000) standard methods for nutrient ascertainment. The moisture value of the *qocho* samples was assessed by the drying air oven (DHG 9055A) at 105°C for an hour by taking about 5.0 g of the sample, following the guidelines in the AOAC (2000) method 925.09. The crude protein analysis was described by the Kjeldahl method (nitrogen value multiplied by 6.25) by taking about 0.5 g of the *qocho* sample according to the AOAC (2000) method 979.09. The analysis of crude fat value was performed by taking about 2.0 g of the *qocho* sample and using the Soxhlet extraction method with diethyl ether as a solvent according to standard procedures 920.39 of the AOAC (2000). The official method 923.03 was employed to ascertain the total ash value following AOAC (2000) by taking 2.5 g of the *qocho* sample. The value of the crude fiber was determined by using the steps of digestion, filtration, washing, drying, and combustion according to standard method 962.09 of the AOAC (2000). The difference from proximate composition was used to determine the total carbohydrate content, and the following formula was used: The total carbohydrate (%) is 100—(% crude protein + % crude fiber + % total ash + % crude fat).

### 2.4 Energy Value Determination

The caloric energy value (kcal/100 g) of *qocho* samples was measured from the contents of crude protein, crude fat, and total carbohydrates employing the following conversion ratios, based on Atwater: (4 kcal/g) for crude protein, (9 kcal/g) for crude fat, and (4 kcal/g) for total carbohydrates (Nguyen et al. 2007). Gross energy is expressed mathematically as follows: 4 × g crude protein + 9 × g crude fat + 4 × g total carbohydrate = Gross energy kcal/100 g.

### 2.5 Determination of Minerals

The mineral concentration of the floured *qocho* samples of eight enset landraces was ignited to ash at 550°C for 5 h in a muffle furnace, dissolved in 20% HCl, and boiled to bring the ash into solution form. The digested *qocho* sample was filtered into an acid‐washed volumetric flask of 100 mL after cooling, and the volume was then adjusted with distilled, deionized water. The mineral contents were determined using calibration curves made using the standard solutions for the minerals. The magnesium, calcium, zinc, and iron values in the sample were measured using an atomic absorption spectrophotometer (Shimadzu, model AA‐6800), while potassium and sodium values were analyzed using flame photometry (Jenway model; pfp7, UK) as per Osborne and Voogt (1978). Phosphorus was determined by using a UV–VIS spectrophotometer (Thermo Scientific model; Evolution 220, USA).

### 2.6 Determination of Anti‐Nutritional Factors

The phytate concentration of the *qocho* samples was analyzed using Latta and Eskin (1980) as modified by Vaintraub and Lapteva (1988). A 1.0 g sample of *qocho* was extracted for 1 h using 10 mL of 0.2 N HCl, and then it was centrifuged for 30 min at 3000 rpm. Wade reagent (2 mL) was added to 3 mL of the supernatant solution, and the combination was centrifuged. An ultraviolet spectrophotometer was applied to measure the absorbance at 500 nm (Lambda 950) (Thermo Scientific model Evolution 220, USA).

The tannin value was measured applying the procedures of Burns (1971), as modified by Maxson and Rooney (1972). After centrifuging at 1000 rpm for 5 min, the produced supernatant solutions from 1 g of *qocho* were combined with 5 mL of vanillin–HCl reagent. Once the reaction was completed, a spectrophotometer set to 500 nm was used to measure the absorbance. The oxalate concentration of the *qocho* was ascertained through the methods indicated by Ukpabi and Ejidoh (1989), which consist of three steps—digestion, oxalate precipitation, and permanganate titration.

### 2.7 Determination of Mineral Bioavailability

The molar ratios between anti‐nutrients and minerals, phytates: calcium, phytates: iron, phytates: zinc, phytates × calcium: zinc, and oxalates: calcium, were used to predict the bioavailability of calcium, iron, and zinc (Norhaizan and Nor Faizadatul Ain 2009).

### 2.8 pH Value and Titratable Acidity Analysis

The pH of the *qocho* was estimated by homogenizing a 1:10 dilution of the sample (10 g of *qocho* samples in 90 mL of distilled H_2_O) and by connecting a glass electrode to a digital pH meter at room temperature after calibrating the pH meter at 7.00, 4.00, and 9.2 with a known buffer solution. The experiment was performed in triplicate.

The sample was titrated with the addition of a standard base (0.1 N NaOH) to 3–5 drops of 1% phenolphthalein indicator until the endpoint, which was identified by the sample changing color to pink. This allowed for the determination of the total titratable acidity of the enset's qocho flour. The equation provided below determines the % titratable acidity based on lactic acid, the predominant organic acid in *qocho* samples, using the volume of NaOH (Karssa et al. 2014).

$$\begin{array}{c} \text{Titratable}\;\text{acidity}\left(\%\right)\;=\;\\ \;\frac{\mathrm{Vol}.\text{NaOH}\;\text{used}\;\left(\mathrm{mL}\right)\;\times \;0.1\;N\;\text{NaOH}\;\times \;0.009\;\times \;100}{W} \end{array}$$

where Vol = volume of the 0.1 N NaOH used, *N* = normality of the NaOH used, 0.009 = milli‐equivalent factor for lactic acid, *W* = weight of the corm sample.

### 2.9 Statistical Analysis

Analyses of variance (ANOVA) were performed using SAS version 9.4 software for Windows to compute data from laboratory tests for the nutritional, anti‐nutritional, molar ratios, and physiochemical contents of traditionally fermented *qocho* derived from eight enset landraces. Duncan's multiple range test procedures were used to compare the means of each parameter among *qocho* of enset landraces, revealing statistically significant differences at a 5% probability level.

## 3 Results and Discussion

### 3.1 Proximate Composition

#### 3.1.1 Moisture Content

The moisture value of the *qocho* of eight enset landraces had varied significantly, with a mean value ranging from 59.03% to 66.50% wet bases (Table 1). The fermented *qocho* sample obtained from the *Anchire* landrace revealed the highest value, whereas the *qocho* samples produced from the *Qiniwara* landraces exhibited the lowest moisture value. The moisture variations between the landraces are explained by the retention potential of water and the type of landraces. However, the moisture contents of *qocho* samples derived from *Agade, Gimbo, Hayiwona*, and *Separa* landraces with the values of 61.21%, 61.23%, 62.02%, and 62.02% on a wet basis, respectively, did not reveal significant variations (*p* > 0.05). Food moisture content is an indicator of food solids content as well as a sign of storage stability (Mauer 2024). Similarly, Punchay et al. (2020) stated that moisture measurement on food products is one of the most typical assessments performed since the moisture content in it has a substantial impact on protection and the existence of physical, chemical, and microbial changes during storage. Accordingly, moisture value is a key component in the fermentation procedure (Adegunloye and Oparinde 2017). The fresh (unfermented) enset pseudostems retain a significant amount of water, ~85%–90% (Nurfeta et al. 2008; Mohammed et al. 2013). The moisture value of the 90‐day traditionally fermented enset *qocho* in the current study is slightly higher than the content (45.50%–60.20%) reported by Yirmaga (2013), and comparable with the reports of Karssa and Papini (2018) (59.90%–63.30%). In the same manner, after 7 weeks of fermentation, the landrace *Addo* retains almost 60% of its moisture content, according to findings by Urga et al. (1997). As *qocho* is fermented for longer, its water content falls, most likely as a result of excessive permeation during pit fermentation (Karssa et al. 2014; Bosha et al. 2016; Andeta et al. 2018). In general, in addition to fermentation time, such a wide range of differences among studies may be attributed to the variety of landraces used, processing methods, environmental factors, and management practices of the enset crops. As stated by Punchay et al. (2020), crops with a high moisture content give coenzymes and water‐soluble enzymes, which are essential for metabolic processes, a bigger role. Furthermore, increased moisture levels promote the enzymatic activity of glycosidase, which aids in the removal of hydrogen cyanide (HCN) content (Safdar et al. 2020).

**TABLE 1:** Proximate composition (%) and gross energy (kcal/100 g) of *qocho* produced from eight selected enset landraces.

Landrace	Moisture^1^	Total ash	Crude protein	Crude fat	Crude fiber	Total CHO	Gross energy
*Agade*	61.21^c^	1.22^c^	1.75^d^	0.33^d^	2.79^cd^	93.93^b^	385.69^bc^
*Anchire*	66.50^a^	1.61^b^	2.28^b^	0.26^f^	4.85^b^	91.01^e^	375.51^d^
*Gimbo*	61.23^c^	1.23^c^	3.15^a^	0.68^b^	2.85^c^	92.12^d^	387.20^ab^
*Hayiwona*	62.02^c^	1.20^c^	2.10^bc^	0.73^a^	2.71^d^	93.26^c^	387.97^a^
*Hiniba*	64.20^b^	1.21^c^	1.93^cd^	0.28^ef^	2.25^f^	94.34^a^	387.63^a^
*Qiniwara*	59.03^d^	1.23^c^	1.75^d^	0.14^g^	2.27^f^	94.64^a^	386.82^ab^
*Separa*	62.02^c^	1.61^b^	2.28^b^	0.31^de^	2.42^e^	93.39^c^	385.12^c^
*Sisqella*	62.52^bc^	2.42^a^	2.10^bc^	0.37^c^	5.39^a^	89.74^f^	370.69^e^
MSE±	1.08	0.19	0.12	0.02	0.05	0.19	0.23
CV	1.73	13.13	5.34	5.69	1.59	0.21	0.89

*Note:* Mean values are the results of the triple analysis, and the values in a column with different superscripts indicate significant differences at the *p* < 0.05 level. 1 = wet basis.

Abbreviations: CHO = carbohydrate, CV = coefficient of variance.

#### 3.1.2 Protein Content

Table 1 summarizes the mean crude protein value of *qocho* samples taken from eight distinct enset landraces. The mean value of crude protein content of the *qocho* samples examined varied between 1.75% and 3.15%. The results of the present study indicated that the protein content of the enset *qocho* under study varied significantly among landraces. On a dry weight basis, the maximum crude protein content was derived in the *qocho* sample of the enset landrace *Gimbo* (3.15%), followed by *Anchire* (2.28%) and *Separa* (2.28%), while the lowest content was recorded in the *qocho* samples produced from *Agade and Qiniwara* (1.75%) for each landrace. These values were higher even compared with the amounts reported by Atlabachew (2007), 1.1%–2.8%, Bosha et al. (2016), 1.54%, and Tuffa (2019), 1.59%–1.67%, but our present result was closely similar to the results of Urga et al. (1997), 3.47% from *Addo* landrace, Forsido et al. (2013), 2.57% from *Nobo* landrace, and Karssa and Papini (2018), 2.97%–3.53%. In contrast, Yirmaga (2013) reported 5.09% protein at 30 days of fermentation. The protein content varies within and among the studied *qocho* samples of different enset landraces. This disparity could be due to genotypic differences among the selected enset plants, fermentation time, and age of the plants. The protein contents of the crops and their nourishing could be beneficial for healing and replacing body tissues. Moreover, enset farmers assert that the primary food products of some enset landraces are essential for human bodybuilding and motivating cows to produce milk after birth (Dilebo, Feyissa, Asfaw, and Zewdu 2023a). Dietary proteins are necessary for the natural production and upkeep of body tissues, hormones, enzymes, and other chemicals needed for normal functioning (Bender and Cunningham 2021).

#### 3.1.3 Total Ash Content

The mean total ash concentrations from the produced *qocho* samples had varied significantly, ranging from 1.20% to 2.42% (Table 1). *Qocho* produced from the *Sisqella* landrace (2.42%) had the maximum total ash value, followed by *Anchire* and *Separa* (1.61%) for each landrace. However, the *qocho* sample produced by *Hayiwona* had the lowest total ash concentration, measuring 1.20%. Similarly, *qocho* samples obtained from *Agade*, *Gimbo*, *Hayiwona*, *Hiniba*, and *Qiniwara* landraces exhibited a closely equal amount of total ash value and did not show significant differences (*p* > 0.05) (Table 1). In the present study, the total ash value of the *qocho* of *Sisqella* enset landraces became nearly twofold in comparison with the *Agade*, *Gimbo*, *Hayiwona*, *Hiniba*, and *Qiniwara* varieties. The mean value of the total ash concentration observed in the current study is nearly similar to the reports of Atlabachew (2007), Forsido et al. (2013), Yirmaga (2013), Karssa and Papini (2018), and Tuffa (2019); it varied from 1.25 to 2.35 g/100 g. On the other hand, the lowest total ash values (0.63%) and the highest content (4.47%) were reported by Urga et al. (1997) and Bosha et al. (2016), respectively. The observed variations could be explained by factors such as the age of the enset crop, fermentation length, agronomic methods, genetic makeup, and environment. Similarly, Bosha et al. (2016) and Karssa and Papini (2018) reported that fermentation impacts the solubility of mineral elements and organic components of the *qocho*. According to Allai et al. (2022), the total mineral content of food is represented by its ash content, which is important for its physicochemical and nutritional value while making up a minor amount of dry substance. The *qocho* of some enset landraces revealed comparable ash values with other traditionally or naturally fermented roots and tuber crops and a greater amount with the majority of grains. This indicates that the better ash value of *qocho* means enset crops have the ability to increase dietary mineral contents, which benefits consumers' health directly. This could be a reason for the traditional recommendation from farmers that certain bone fractures and muscle‐related issues in humans, as well as cattle, can be treated with *qocho* derived from some enset landraces (Tsehay and Kebebew 2006; Olango et al. 2014; Dilebo, Feyissa, Asfaw, and Zewdu 2023a). According to Gharibzahedi and Jafari (2017), plentiful mineral contents in diets promote growth and development and speed up biochemical roles in the body.

#### 3.1.4 Crude Fat Content

The results showed a significant variation (*p* < 0.05) in the crude fat value of *qocho* samples derived from the eight enset landraces. As given in Table 1, all the *qocho* samples had low crude fat concentrations, falling below 1.0%, and varied from 0.14% to 0.73%. The *qocho* sample of the *Hayiwona* landrace had a comparatively higher crude fat level of 0.73%, followed by *Gimbo* (0.68%). The recorded values in the present study are slightly comparable with the reports of Urga et al. (1997) (0.43%), Bosha et al. (2016) (0.45%), and Tuffa (2019) (0.2%). However, a relatively higher crude fat value in the range of 1.04%–1.39% was reported by Forsido et al. (2013) and Yirmaga (2013). The observed variations can result from genetics, aging, environmental variables, or the harvesting season of the enset crops. In addition to giving foods energy value, fat aids in the body's physiological functions, such as digestion, absorption, and the transportation of fat‐soluble vitamins (Yimer et al. 2023). According to Azeredo et al. (2021), dietary fats increase food's palatability by absorbing and retaining flavors, which is very important from the nutritional point of view because 1 g of fat gives 9 kcal energy. Low‐fat values in food samples potentially contribute to increased shelf duration due to decreased possibilities of rancidity (Adejuwon et al. 2020). However, excess crude fat intake has well‐established health implications in humans (Hammad et al. 2016).

#### 3.1.5 Crude Fiber Content

The mean crude fiber value in the fermented *qocho* samples of the eight enset genotypes was displayed in Table 1 and has a significant variation (*p* < 0.05), which ranges from 2.25% to 5.39%. The *qocho* sample derived from the *Sisqella* landrace had the highest crude fiber percentage (5.39%), followed by the *Anchire* landrace (4.85%), while the *Hiniba* landrace (2.25%) had the lowest crude fiber content. Based on a dry weight basis, crude fiber was the second highest proximate composition observed in the *qocho* samples in the present study, below the total carbohydrate value. The fiber value found in the present study was consistent with reports from Urga et al. (1997), Bosha et al. (2016), and Karssa and Papini (2018), who reported that the fiber content of enset landrace *qocho* in Ethiopia ranged from 3.22% to 5%. In contrast, a lower crude fiber content of 1.89% was reported by Yirmaga (2013) from the *qocho* samples of the *Astare* and *Kinnare* landraces at 30 days of fermentation. The age of the plant, genotype, and the duration of fermentation may all contribute to this wide range of values. According to our results and the literature, some enset *qocho* has higher contents and a noteworthy amount of crude fiber when compared with other root crops and tubers, such as anchote, cassava, and Irish potatoes (Parmar et al. 2017). Crude fiber is necessary for proper digestion and waste elimination from the body (Maćkowiak et al. 2016; Hussain et al. 2020). In a similar vein, Millar et al. (2019) described that fiber keeps regular bowel processes and can avoid some gastrointestinal ailments. Crude fiber serves as a component of food and is becoming considered a useful tool in regulating oxidative processes in food products (Barber et al. 2020). Furthermore, dietary fiber reduces blood cholesterol and the risk of a number of ailments in humans (Jacob et al. 2015; Millar et al. 2019); it is also essential for the protection against colorectal cancer, obesity, and constipation (Biswas et al. 2020).

#### 3.1.6 Total Carbohydrate Content

The total carbohydrate value on a dry basis for each of the eight enset genotypes is displayed in Table 1 and is significantly different (*p* < 0.05). The mean total carbohydrate values of fermented *qocho* samples varied from 89.74% for the *Sisqella* landrace to 94.64% for the *Qiniwara* landraces. Nonetheless, there were no statistically significant variations (*p* > 0.05) in the total carbohydrate values of the *Hayiwona* and *Separa* landraces, which were 93.26% and 93.39% on a dry weight basis, respectively. Similarly, in the *qocho* samples of the landraces *Hiniba* and *Qiniwara* enset, there was no statistically significant variation (*p* > 0.05) found for the total carbohydrate (%). Yirmaga (2013) notified the total carbohydrate values (33.8%) of the 30 days fermented *qocho* samples of *Astare* and *Kinnare* enset varieties; this is significantly less than the values of carbohydrates overall found in enset *qocho* in the present study. The observed notable discrepancies among the total carbohydrates of the examined *qocho* samples may be attributed to distinct compositions of crude protein, total ash, crude fat, and crude fiber, as well as the fermentation time and processes. Nonetheless, the current value is slightly similar to the reports of Atlabachew (2007). *Qocho* has nutritional contents similar to potatoes (Tsegaye and Struik 2001; Mohammed et al. 2013), which are rich in carbohydrates (Andeta et al. 2018; Tuffa 2019). The present study's laboratory results also demonstrated that the *qocho* samples obtained from each genotype exhibited a high amount of carbohydrate value, lending acceptance to the notion that root crops are generally opulent in carbohydrates. In the same manner, Padhan et al. (2020) reported that the dry matter content of most root crops ranges from 60% to 90% carbohydrate. Likewise, the study's results on the carbohydrate value of *qocho* were roughly similar to results documented in other root and tuber crops in Ethiopia.

### 3.2 Gross Energy Value

As presented in Table 1, the energy content (kcal/100 g) of the fermented *qocho* samples obtained from the eight genotypes differed significantly (*p* < 0.05), ranging from 370.69 to 387.97. Fermented *qocho* samples derived from the *Hayiwona* and *Sisqella* enset landraces had the maximum and minimum total energy contents, respectively. *Qocho* is widely recognized for having high energy content (Pijls et al. 1995; Tsegaye and Struik 2001; Andeta et al. 2018). The body needs food based on its energy content, which measures the chemical energy contained in the food's organic substance bonds, including its fat, protein, and carbohydrates components, according to Lowe (2021). In general, from a nutritional perspective, root crops are excellent sources of dietary energy (Devaux et al. 2021). The energy values reported by Daba and Shigeta (2016) for the *bulla* sample of the *Neqaqa* enset landrace (363 kcal/100 g) are lower than the values of the enset *qocho* samples observed in this study. In contrast, a higher energy content of 393.19–394.24 was reported by Bekele et al. (2023) from four landraces at 30 days of the fermented *bulla*, another enset product. The difference could be explained by variations in management, environment, genetics, and the enset product utilized. Nonetheless, the energy values of enset's *qocho* that were found in the current study were slightly in line with those found in other roots and tubers that are high in energy. As a result, significant levels of energy content were found in the *qocho* samples derived from each landrace. This indicates that enset *qocho* is a valuable and the most affordable energy source for many people.

### 3.3 Mineral Content

Table 2 presents the average mineral composition (Ca, K, Mg, P, Na, Fe, and Zn) of fermented *qocho* samples from eight chosen enset genotypes. Statistically significant differences were observed in the mean calcium values among the examined *qocho* of enset landraces, which ranged from 80.17 mg/100 g for *Hayiwona* to 110.60 mg/100 g for *Anchire* and 110.52 mg/100 g for *Sisqella* genotypes. Tuffa (2019) reported the calcium values of two enset landraces *qocho* from 88.00 to 108.88 mg/100 g; it is within the range of what we found in this study. On the other hand, Urga et al. (1997) and Bosha et al. (2016) reported greater calcium content (142.3 mg/100 g) and (179.92 mg/100 g), respectively. Conversely, lower calcium concentrations of *qocho* from various enset landraces, ranging from 48.90 to 58.40 mg/100 g, were reported by Atlabachew (2007). The difference could result from management practices, genetics, or the environment. Likewise, the calcium concentrations in this study were closely similar to those Parmar et al. (2017) published prior to for other common crops derived from roots and tubers. Calcium is essential for bone composition, tooth formation, and blood clotting in humans (Jacob et al. 2015; Tedeschi et al. 2023), and calcium also maintains and performs the biological functions of glandular production, muscular contraction, and nerve transmission (Kiczorowski et al. 2022).

**TABLE 2:** Mineral contents (mg/100 g) of *qocho* produced from eight enset landraces.

Landrace	Calcium	Potassium	Magnesium	Phosphorus	Sodium	Iron	Zinc
*Agade*	90.26^e^	107.08^d^	15.61^ab^	40.19^a^	7.41^c^	5.20^e^	0.68^b^
*Anchire*	110.60^a^	157.14^a^	15.43^ab^	30.33^c^	8.35^a^	4.26^f^	0.47^e^
*Gimbo*	100.63^c^	127.11^c^	14.86^b^	40.17^a^	7.62^bc^	6.71^a^	0.41^f^
*Hayiwona*	80.17^g^	90.55^e^	16.27^a^	10.84^g^	7.43^c^	5.43^d^	0.73^a^
*Hiniba*	90.93^d^	110.72^d^	15.32^ab^	20.94^e^	7.78^abc^	5.45^d^	0.60^c^
*Qiniwara*	80.96^f^	137.06^b^	16.35^a^	30.54^b^	8.09^ab^	6.33^c^	0.39^f^
*Separa*	100.64^c^	90.35^e^	14.99^b^	30.02^d^	7.59^bc^	6.50^b^	0.46^e^
*Sisqella*	110.42^b^	150.53^a^	14.37^b^	20.72^f^	7.84^abc^	4.08^g^	0.51^d^
MSE±	0.03	5.29	0.67	0.04	0.31	0.02	0.02
CV	0.04	4.36	4.35	0.13	4.04	0.37	2.74

*Note:* Mean values are the results of the triple analysis, and the values in a column with different superscripts indicate significant differences at the *p* < 0.05 level.

Abbreviation: CV = coefficient of variance.

The potassium content of fermented *qocho* samples from eight enset landraces varied significantly, with a mean value ranging from 90.35 to 157.14 mg/100 g on a dry weight basis (Table 2). The potassium concentration in fermented *qocho* samples from the *Anchire* landrace was found to be the greatest, whereas those from the *Separa* landrace had the lowest value. The present finding was indicated by the far lower potassium values of fermented *qocho* samples reported by Atlabachew (2007) and Tuffa (2019), which ranged from 275.30 to 482.30 mg/100 g. In contrast, Bosha et al. (2016) described that the fermented enset *qocho* of various genotypes had a much lower potassium concentration (63.92 mg/100 g). Such greater differences appeared to be caused by the length of the fermentation process and duration, the climate, types of soil, and enset crop varieties employed in the experiment. Potassium is necessary for maintaining the body's water balance, heart rate, neurotransmission, and ability to manage hypertension (Jacob et al. 2015; Stone and Weaver 2021). Moreover, potassium can improve iron utilization, control osmotic pressure and pH equilibrium, and be helpful to individuals using diuretics to treat hypertension (Gebeyehu et al. 2024).

The mean magnesium value in the *qocho* of the enset varied from 14.37 mg/100 g for *Sisqella* to 16.35 mg/100 g for *Qiniwara*, as presented in Table 2. The analyzed contents revealed no significant variance within the genotypes of *Qiniwara* and *Hayiwona; Agade*, *Anchire*, and *Hiniba; Gimbo, Separa*, and *Sisqella*. The mean magnesium value of *qocho* samples in the present study was by far higher than that observed by Bosha et al. (2016) (1.84 mg/100 g) and lower than the findings of Atlabachew (2007) (18.00–29.00 mg/100 g). However, the value reported by Tuffa (2019) is closely comparable to the present findings. The differences in magnesium concentration observed between our results and those of earlier research could be attributed to variations in soil composition, environmental conditions, and genetic diversities. Likewise, the magnesium levels observed from the current study are in line with Parmar et al. (2017), who reported a similar content for cassava ( *Manihot esculenta* ) and sweet potato ( *Ipomoea batatas* ). Magnesium is essential for the nervous and muscular systems to function properly and for protein metabolism (De Baaij et al. 2015). It also helps to maintain a robust immune system, builds bone density, and facilitates blood sugar regulation, which in turn supports healthy blood pressure (Olatunde et al. 2016). Furthermore, magnesium is necessary for proper metabolism of calcium and phosphorus, muscle contraction, neuronal and membrane electrical potential maintenance, and various enzyme system functioning (Mathew and Panonnummal 2021).

Mineral analysis of the observed fermented *qocho* samples of the different enset genotypes revealed a significant variation (*p* < 0.05) in phosphorus concentration, with the maximum content of 40.19 mg/100 g for *Agade* and 40.17 mg/100 g for *Gimbo qocho* samples documented on a dry basis. Both genotypes offered four times higher phosphorus values than those of *Hayiwona* and twice greater than those of *Hiniba* and *Sisqella* landraces (Table 2). The average content of phosphorus in this study was by far lower than the content described by Urga et al. (1997) (128.30 mg/100 g) after 7 weeks of fermentation of the *qocho* of enset landraces. However, the content reported by Tuffa (2019) is slightly comparable to the present findings from the *Agade* and *Gimbo* enset landraces. It is possible to explain the variation found in both the previous study and the present results by genetic variations among landraces, environmental conditions, soil fertility, agronomic approaches, fermentation time, and the plant age. Phosphorus contributes an essential role in the synthesis of nucleic acid, healthy cell development and repair, and the ossification of bones by being deposited as calcium phosphate (Jeong et al. 2019; Hou et al. 2022). Similarly, Fekadu et al. (2021) and Gebeyehu et al. (2024) described that phosphorus is a crucial constituent of all living cells and plays a part in the enzyme‐regulated energy‐yielding metabolism, which also aids in the controlling of the blood's acid‐alkaline balance.

There was variation in the mean sodium values among the analyzed *qocho* samples of genotypes from a level of 7.41 mg/100 g in *Agade* to 8.35 mg/100 g in *Anchire* (Table 2). However, the measured mean values did not reveal statistically significant variations within the genotypes of *Agade* and *Hayiwona*, *Gimbo* and *Separa*, and *Hiniba* and *Sisqella* regarding sodium concentrations. The findings of the present mineral tests for the *qocho* samples of eight different enset genotypes showed greater sodium concentrations than those found by Bosha et al. (2016) (3.56 mg/100 g) and Tuffa (2019) (2.97 mg/100 g). However, these contents were much lower than the stated values for the corm samples by Forsido et al. (2013) (15 mg/100 g) and by Mohammed et al. (2013) (30 mg/100 g) for the different enset landraces. The variation could be attributed to differences in the enset product used, the growth environment, or genetic makeup. Sodium is the main extracellular ion in the body, regulating bodily fluids and maintaining tissue electric potential (Seifter and Chang 2017). However, consuming excess sodium might raise blood pressure (Grillo et al. 2019; Gebeyehu et al. 2024).

The iron values in the fermented *qocho* samples of enset varied significantly (*p* < 0.05) between the landraces in the present study, ranging from 4.08 mg/100 g in the *Sisqella* landrace to 6.71 mg/100 g in *Gimbo* (Table 2). The mean value of iron in this study is closely similar to the findings of Abebe et al. (2007), Bosha et al. (2016) and Tuffa (2019) for the *qocho* samples of different enset landraces. However, Urga et al. (1997) reported far lower iron concentrations (1.60 mg/100 g) from 7‐week fermented *qocho* samples of *Nobo* landraces. This disparity in iron concentration may be explained by variations in genetic diversity, climate factors, soil composition, and fermentation times of the samples. Important trace elements, such as iron, are needed in tiny amounts by the human body for essential metabolic reactions. Iron is the chief element for the synthesis of hemoglobin, which is vital for the transportation of oxygen throughout the body as well as the oxidation of lipids, proteins, and carbohydrates (Naz et al. 2018; Dixit et al. 2021). In contrast, a persistent iron deficiency can cause hemoglobin content to fall below normal, causing anemia (Murawska et al. 2016).

As presented in Table 2, the mean value of zinc varied significantly (*p* < 0.05) within *qocho* samples of the analyzed enset landraces, and the mean values oscillated from 0.39 to 0.73 mg/100 g. The mean zinc content of the two landraces was highest in *Hayiwona* and lowest in *Qiniwara*. Tuffa (2019) also reported the zinc values (0.73 mg/100 g), which are in line with the value in this study. In contrast, a greater mean zinc value that varied from 2.28 to 3.20 mg/100 g was notified by Atlabachew (2007) and Bosha et al. (2016); this could be attributed to several variables, including genetics, agronomic practices, environmental changes, and the fermentation period of the used samples. Similarly, Ratsavong et al. (2020) reported that the mineral concentration of plants differs significantly based on several aspects such as disease, multiple transferring, sanitation, genotypes, and soil pollution. Zinc is an essential trace mineral that is important for several cellular processes, such as normal growth, brain development, behavioral response, bone formation, and wound recovery (Mlitan et al. 2014). Zinc is also crucial for healthy skin, strong immune function, and resistance to infection (Chasapis et al. 2020). Furthermore, it is crucial for the synthesis of DNA proteins, insulin function, liver activity, the metabolism of the ovaries, testes, and cell division (Chasapis et al. 2020; Vickram et al. 2021). Overall, due to their numerous physiological and metabolic functions, minerals are essential components of the human diet (Hossain et al. 2021).

### 3.4 Anti‐Nutritional Factors

Natural feedstuffs contain chemical compounds called anti‐nutritional factors, or anti‐nutrients, which are produced by plants' regular metabolism (Vikram et al. 2020). Depending on the amount consumed, some of these plant compounds may be advantageous or detrimental to the health of humans and animals (Sinha and Khare 2017). Prior researches on anti‐nutrient contents (e.g., oxalate, tannin, and phytate) on the fermented enset *qocho* have not been performed so far, except Urga et al. (1997), Abebe et al. (2007) described for unmentioned enset phytate value from the Sidama region, and Forsido et al. (2013) for *Nobo* genotype total phenolic values. The phytate, tannin, and oxalate values of the 90 days fermented *qocho* samples of the eight enset genotypes (*Agade*, *Anchire, Gimbo*, *Hayiwona*, *Hiniba*, *Qiniwara, Separa*, and *Sisqella*) are presented in Table 3. The mean phytate contents of the evaluated enset genotypes were statistically varied (*p* < 0.05) from one another, which ranged from 74.28 to 141.19 mg/100 g for *qocho* produced from *Qiniwara* and *Sisqella* genotypes, respectively, as presented in Table 3. The *qocho* sample of *Sisqella* and *Anchire* landraces provided twice higher phytate contents than that of *Qiniwara*. These concentrations are far higher (7 mg/100 g) than those reported by Abebe et al. (2007) for the fermented *qocho* samples. Genetic diversity, environmental factors, fermentation duration, and analytical procedures all seem to have contributed to this great variety between studies. In comparison to the phytate concentrations of *qocho* in the present study, Bekele et al. (2023) showed that the fermented *bulla* values of four enset cultivars were between 9.72 mg/100 g and 14.70 mg/100 g. This suggests that the phytate values of the diverse enset products can vary. Chandrasekara and Kumar (2016) state that a crop's phytic acid content might vary depending on its genetic makeup, environmental factors, and agricultural practices. Phytate is the main inhibitor of mineral uptake in plant‐based foods. Furthermore, the formation of insoluble mineral chelates in the body and food reduces the bioavailability of dietary minerals (Zohora et al. 2018). Phytate contents should therefore be reduced to less than 200 mg/100 g DM in order to lessen the detrimental effects of phytates on the uptake of minerals (Hurrell 2004).

**TABLE 3:** Anti‐nutritional factors (mg/100 g) of *qocho* of eight enset landraces.

Landrace	Phytate	Tannin	Oxalate
*Agade*	97.18^c^	21.67^b^	7.11^e^
*Anchire*	139.51^a^	5.04^h^	8.53^c^
*Gimbo*	90.28^d^	16.65^d^	6.54^f^
*Hayiwona*	125.25^b^	32.05^a^	9.39^a^
*Hiniba*	85.16^e^	9.86^f^	6.54^f^
*Qiniwara*	74.28^f^	17.86^c^	9.10^b^
*Separa*	84.24^e^	11.89^e^	6.26^g^
*Sisqella*	141.19^a^	5.56^g^	8.25^d^
MSE±	1.11	0.12	0.13
CV	1.06	0.76	1.66

*Note:* Mean values are the results of the triple analysis, and the values in a column with different superscripts indicate significant differences at the *p* < 0.05 level.

Abbreviation: CV = coefficient of variance.

The mean tannin content values in fermented *qocho* samples statistically varied (*p* < 0.05) across eight enset genotypes, ranging from 5.04 to 32.05 mg/100 g. Relatively, *Hayiwona* landrace had the greatest tannin content (32.05 mg/100 g), followed by the *Agade* landrace (21.67 mg/100 g) but the *qocho* sample of the *Anchire* landrace, which has the lowest tannin content (5.04 mg/100 g) (Table 3). In contrast to the current study, the tannin level for the enset landrace's 7‐week fermented *qocho* (333 mg/100 g) notified by Urga et al. (1997) was excessively high on a dry basis. These differences could be due to fermentation time, genotype, and age variation in the enset plant and analytical approaches. Tannin is a water‐soluble, high molecular weight molecule that significantly alters nutritional values. Foods high in tannin are considered to have low nutritional value since they can precipitate proteins and inhibit the function of enzymes involved in digestion and reabsorption (Jacob et al. 2015). It is acceptable for humans to consume up to 560 mg of tannin per day (WHO 2003; Ajebli and Eddouks 2019). Consequently, all enset *qocho* samples showed minimal tannin concentrations and are acceptable for intake by humans.

In this study, the mean value of oxalate found from *qocho* samples of eight different enset landraces varied from 6.26 to 9.39 mg/100 g and significantly differed (*p* < 0.05) among enset landraces. The *qocho* sample of the *Hayiwona* landrace had a high oxalate level (9.39 mg/100 g), but the *Separa* landrace sample had lower levels (6.26 mg/100 g) on the basis of dry weight. In contrast, these contents are too low compared with the contents mentioned by Urga et al. (1997) for oxalate (220 mg/100 g) for the *qocho* sample of 7‐week fermented enset landraces. These values are lower than those for other documented Ethiopian root and tuber crops. Diets rich in oxalate can be harmful to human nutrition and health, especially as they hinder the absorption of calcium and contribute to the formation of kidney stones (Jacob et al. 2015; Millar et al. 2019; Mitchell et al. 2019). The results of this study indicate that the levels of oxalate in *qocho* are not high enough to be harmful to human health.

### 3.5 Molar Ratios and Bioavailability of Minerals

The analysis of the anti‐nutrient inhibitory effect on the bioavailability of minerals in food and diet is done using the molar ratios of anti‐nutrients to minerals (Castro‐Alba et al. 2019). The percentage of a consumed nutrient—especially minerals—that is absorbed and used through regular metabolic pathways is known as its bioavailability (Gibson et al. 2010). It is affected by the occurrence of phytates, tannins, oxalates, and dietary fiber (Frossard et al. 2000). Because of this, the gastrointestinal tract will only absorb a minimal amount of the minerals in the consumed food (Norhaizan and Nor Faizadatul Ain 2009). As the molar ratios of phytate to calcium, iron, and zinc exceed 0.24, 1, and 15, respectively, these minerals will be degraded and show evidence of low bioavailability (Ma et al. 2007; Norhaizan and Nor Faizadatul Ain 2009).

There were significant differences (*p* < 0.05) in the amounts of phytates to calcium, iron, zinc, and phytates × calcium to zinc in the fermented *qocho* samples from eight enset landraces. The results for each landrace are displayed in Table 4. The landrace *Hayiwona* had the highest phytate‐to‐calcium molar ratios, reaching 0.10. Conversely, the landraces *Gimbo* and *Separa* showed the lowest values, each at 0.05. In this study, the mean molar ratio of phytate to calcium of all the calculated *qocho* samples of enset landraces was lower than the critical values (< 0.24) (Table 4). This implies that phytates could have no impact on the dietary constituents of the *qocho* samples of the examined enset landraces and showed good calcium bioavailability, which is a result of the high calcium concentration of enset products.

**TABLE 4:** Calculated molar ratios of phytate to Ca, Fe, and Zn and oxalate to Ca of *qocho* from eight enset landraces.

Landrace	Phytate:Ca	Phytate:Fe	Phytate:Zn	Oxalate:Ca	[phytate][Ca]/[Zn]
*Agade*	0.07^c^	1.58^d^	14.13^g^	0.04^b^	33.18^g^
*Anchire*	0.08^b^	2.77^b^	29.35^a^	0.04^b^	83.35^a^
*Gimbo*	0.05^e^	1.14^f^	21.85^c^	0.03^b^	57.45^c^
*Hayiwona*	0.10^a^	1.95^c^	17.02^f^	0.07^a^	34.55^f^
*Hiniba*	0.06^d^	1.32^e^	14.06^h^	0.03^b^	29.32^h^
*Qiniwara*	0.06^d^	0.99^g^	18.94^d^	0.05^ab^	38.12^e^
*Separa*	0.05^e^	1.10^f^	18.19^e^	0.03^b^	46.01^d^
*Sisqella*	0.08^b^	2.93^a^	27.43^b^	0.03^b^	73.83^b^
MSE±	0.002	0.038	0.015	0.013	0.02
CV	2.38	2.23	0.07	31.87	0.04

*Note:* Mean values are the results of the triple analysis, and the values in a column with different superscripts indicate significant differences at the *p* < 0.05 level.

Abbreviation: CV = coefficient of variance.

The *qocho* sample prepared from the *Sisqella* landrace was found to have the greatest phytate to iron (2.93) molar ratios, while the *Qiniwara* landraces had the lowest (0.99). This result showed that the fermented *qocho* samples' phytate to iron molar ratios were higher than the critical value, with the exception of the *qocho* of *Qiniwara* landraces. This suggests that phytate inhibited the absorption of iron from *qocho* samples, and as a result, the high molar ratio content in the *qocho* samples tested may have inhibited the bioavailability of iron. Iron bioavailability is considered to be low when the phytate to iron molar ratio is higher than 1 (Gibson et al. 2018).

The mean phytate to zinc ratios of the landraces *Hiniba* and *Anchire* showed the lowest (14.06) and highest (29.35) values. Based on this result, the *qocho* sample obtained from *Hiniba* and *Agade* enset landraces indicated good zinc bioavailability. Nevertheless, the molar ratios of the remaining landraces were higher than the threshold values (> 15) (Table 4). This implies that *qocho* of different enset landraces has different anti‐nutritional and mineral compositions. A Phytate to zinc molar ratio greater than the threshold (> 15) signifies low zinc bioavailability (Gibson et al. 2018).

The phytate × calcium: zinc molar ratio of the tested *qocho* samples of enset landraces showed a significant difference (*p* < 0.05); the *Anchire* (83.35) and *Hiniba* (29.32) landraces had the highest and lowest ratios, respectively (Table 4). The mean values found in this study were lower than critical values, which indicated good zinc bioavailability. Hence, the phytate × calcium to zinc molar ratio in the fermented *qocho* samples of the analyzed enset landraces was lower than 200, which shows good zinc bioavailability (Norhaizan and Nor Faizadatul Ain 2009). In comparison to the phytate to zinc molar ratio alone, phytate × calcium to zinc provides a more accurate measure of zinc bioavailability (Gibson et al. 2018).

The mean value of the oxalate to calcium molar ratios of the fermented *qocho* samples produced from eight different enset landraces varied from 0.03 to 0.07. Relatively, the highest mean content was found in landrace *Hayiwona* with a molar ratio value of 0.07% (Table 5). This suggests that oxalates may have no impact on the bioavailability of calcium in the analyzed *qocho* samples. This is due to the high calcium content in *qocho* or the low amount of oxalate. The oxalate to calcium molar ratio below the critical value (1) is an indication of good calcium bioavailability (Bhandari and Kawabata 2004).

**TABLE 5:** The pH value and titratable acidity (%) of *qocho* obtained from eight different enset landraces.

Landrace	pH value	TTA%
*Agade*	3.85^b^	1.17^b^
*Anchire*	3.87^b^	1.14c
*Gimbo*	3.86^b^	1.13^cd^
*Hayiwona*	3.92^ab^	1.09^e^
*Hiniba*	3.86^b^	1.13^cd^
*Qiniwara*	3.93^ab^	1.11^de^
*Separa*	3.62^c^	1.21^a^
*Sisqella*	4.05^a^	1.02^f^
MSE±	0.08	0.01
CV	1.95	1.27

*Note:* Mean values are the results of the triple analysis, and the values in a column with different superscripts indicate significant differences at the *p* < 0.05 level.

Abbreviation: CV = coefficient of variance.

### 3.6 pH and Total Titratable Acidity

Table 5 shows the pH and total titratable acidity value of the *qocho* samples obtained from the eight enset landraces. The *qocho* samples from *Anchire* landraces had the greatest mean pH value (4.05) among the examined enset landraces, while *Separa* landraces had the lowest pH value (3.62). Similarly, comparable mean pH values were reported by Urga et al. (1997) (3.80) and by Yirmaga (2013) (3.79) for the fermented *qocho* samples from different types of enset landraces. This confirms that the fermented *qocho* samples used in the present study had acidic pH values, which satisfied quality standards and were considered acceptable. The pH is a significant intrinsic component in fermented food since it establishes the microbiological stability against pathogenic microbes and food deterioration, in addition to being linked to the flavor of the product (Batista et al. 2019). Food samples that have a pH of 4 or below would exhibit a typically sour taste and odor due to fermentation (Li et al. 2015); the pH value is an appropriate quality determiner for naturally fermented foods (Sharma et al. 2020).

Table 5 shows that there was a significant variation in the mean percentage of total titratable acidity among the analyzed *qocho* samples of the enset landraces in the present study. The *qocho* sample derived from *Separa* had the highest mean titratable acidity value (1.21%), while the *Sisqella* landraces had the lowest mean titratable acidity percent (1.02%). Urga et al. (1997) reported the titratable acidity value of a 7‐week fermented *qocho* sample (1.54%), which was slightly higher than the *qocho* of enset titratable acidity in the present study. Conversely, the results of our findings are higher than the titratable acidity notified by Yirmaga (2013) (0.87%) for the 30‐day fermented enset *qocho* samples. The differences could be attributed to the genotypic, environmental, and length of the fermentation of the *qocho* samples.

## 4 Conclusion

This study has revealed significant differences in the nutritional and anti‐nutritional contents of fermented *qocho* samples produced from the eight widely cultivated enset landraces. As compared to other examined landraces, the *qocho* samples of *Sisqella, Anchire*, and *Hayiwona* exhibited the highest nutritional parameters, followed by *Gimbo* in the present study. The present results also indicate that all of the considered *qocho* samples of enset genotypes have lower levels of anti‐nutritional components; it is favorable in terms of nutrition. In the same manner, the molar ratios of the *qocho* in the present finding were below the critical levels for phytate to calcium, phytate × calcium to zinc, and oxalate to calcium, which show good mineral bioavailability. In contrast, the molar ratios of the *qocho* samples in the present study were beyond the critical levels for phytate to iron and phytate to zinc, which show poor mineral bioavailability. However, *qocho* is usually consumed after being baked with milk products and vegetables, which increases the mineral level and improves the uptake of nutrients from the enset food. Thus, the present findings provide useful information on the nutritional and anti‐nutritional contents of *qocho*, which are significantly varied due to genotypic differences. It could be an excellent, low‐cost source of food diversification. Therefore, sustainable conservation methods and further expansion to new areas and regions of the country are critical in order to ensure food and nutrition security.

## Author Contributions

**Tesfaye Dilebo:** conceptualization (equal), data curation (equal), formal analysis (equal), investigation (equal), methodology (equal), resources (equal), software (equal), visualization (equal), writing – original draft (equal), writing – review and editing (equal). **Ashagire Zewdu:** conceptualization (equal), data curation (equal), methodology (equal), resources (equal), supervision (equal), validation (equal), writing – review and editing (equal).

## Ethics Statement

The authors have nothing to report.

## Conflicts of Interest

The authors declare no conflicts of interest.

## Data Availability

Data will be made available on request.
